# Evaluation of a Trauma Pathway Within an Increasing Access to Psychological Therapies (IAPT) Service

**DOI:** 10.1192/bjo.2022.308

**Published:** 2022-06-20

**Authors:** Grace Jell, Diane Kohl, Alison Salvadori, Annabel Beasley, Tai Ken Ting, Henry Collier, Shruti Dholakia, Verity Bushell, Vijay Gill, Katherine Beck

**Affiliations:** 1 Berkshire Healthcare NHS Foundation Trust, Reading, United Kingdom; 2 Berkshire Healthcare NHS Foundation Trust, Maidenhead, United Kingdom; 3 33N, London, United Kingdom.; 4 Imperial College Hospital NHS Trust, London, United Kingdom; 5 Northern Care Alliance NHS Foundation Trust, Salford, United Kingdom; 6 Guy's and St Thomas' NHS Foundation Trust, London, United Kingdom; 7 South West London and St Georges Mental Health NHS Trust, London, United Kingdom

## Abstract

**Aims:**

The Enhanced Trauma Pathway (ETP) at Berkshire Healthcare NHS Foundation Trust was established in 2018 to manage high demand on a highly specialist psychology team called the Berkshire Traumatic Stress Service (BTSS). The ETP is used to treat complicated cases of Post-Traumatic Stress Disorder (PTSD) within the IAPT service. However, because of the ETP there is now a cohort of Service Users (SUs) presenting to IAPT with a higher complexity than has been typical, presenting new challenges for the service. We aim to evaluate and redesign the ETP within IAPT to meet the needs of the changing population.

**Methods:**

Clinically Led workforcE and Activity Redesign (CLEAR) is a workforce transformation methodology with four unique stages: i) Clinical Engagement: in-depth qualitative analysis of interview data from staff ii) Data Interrogation: cohort analysis using clinical and workforce data visualisations and analysis, iii) Innovation: developing novel solutions with insights from triangulated qualitative and quantitative data, iv) Recommendations: formulation of new models of care (NMOC) and smaller quick high impact service innovations. Thematic analysis was used for the qualitative data. Quantitative data analysis was conducted using the IAPT dataset.

**Results:**

27 semi-structured interviews were conducted with staff. SUs on the ETP had longer waiting times, their treatment took longer (18 sessions for ETP Vs 12 for core step 3) and they had lower recovery rates: 32.9% for ETP, 49.9% for core step 3 in IAPT and 57.3% for the whole IAPT service. SUs on the ETP presented with increased risk concerns, often not mitigated by stabilisation work offered. Thematic analysis also identified challenges with recruitment, a lack of qualified staff and inefficient use of skills across the pathway. Staff well-being was found to be paramount, however supporting staff was found to be challenging due to national constraints placed upon IAPT and the targets the service is asked to achieve. A series of recommendations were made including three options for a NMOC. The options suggested different ways to redesign the pathway including an option where there would be a trauma only team within IAPT working exclusively on the ETP.

**Conclusion:**

This evaluation highlights the challenges for the ETP and identifies NMOC to reduce their impact on the service. Further work is required to assess the NMOC once it has been implemented and to further evaluate the needs of the SUs presenting to this service.

